# Models predicting the risks of six life-threatening morbidities and bile leakage in 14,970 hepatectomy patients registered in the National Clinical Database of Japan: Erratum

**DOI:** 10.1097/MD.0000000000006813

**Published:** 2017-04-28

In the article, “Models predicting the risks of six life-threatening morbidities and bile leakage in 14,970 hepatectomy patients registered in the National Clinical Database of Japan”,^[1]^ which appeared in Volume 95, Issue 49 of *Medicine*, affiliation C appeared incorrectly as “National Clinical Database, Sapporo, Tokyo, Japan.” It has been changed to appear as “National Clinical Database, Tokyo, Japan.”

In Figure 1 section B, the numbers 0.635 and 0.601 appeared in red font when they should have been black. This has since been corrected.
